# Simultaneous Functional and Morphological Assessment of Left Atrial Appendage by 3D Virtual Models

**DOI:** 10.1155/2019/7095845

**Published:** 2019-05-21

**Authors:** Giorgia Vivoli, Emanuele Gasparotti, Marco Rezzaghi, Elisa Cerone, Massimiliano Mariani, Luigi Landini, Sergio Berti, Vincenzo Positano, Simona Celi

**Affiliations:** ^1^BioCardioLab, Fondazione CNR–Regione Toscana “G. Monasterio”, Massa, Italy; ^2^Department of Interventional Cardiology, Fondazione CNR–Regione Toscana “G. Monasterio”, Massa, Italy; ^3^Department of Information Engineering, University of Pisa, Pisa, Italy

## Abstract

**Purpose:**

The left atrial appendage (LAA) is responsible for thrombus formation in patients with atrial fibrillation. The evaluation of both LAA function and morphology is crucial for the patient characterization and the preprocedural planning of LAA closure intervention. Despite the availability of 3D imaging modalities, the current standard image analysis is based on manual delineation of the LAA contours on 2D views.

**Methods:**

In this study, a comprehensive approach based on a full 3D analysis of the tomographic dataset by surface extraction and processing (3D-S) is presented. The proposed method allows extracting functional and morphologic information in the entire cardiac cycle by minimalizing manual user interaction. The proposed methodology has been validated on ten computer tomography datasets.

**Results:**

The proposed 3D-S method was feasible in all cases. Reproducibility was improved with respect to the reference 2D manual procedure (2D-S) (coefficient of variation 2.9 vs. 4.1% for diastolic ostium area; 3.8 vs. 6.1% for systolic ostium area; 2.4 vs. 5.3% for diastolic LAA volume; 2.7 vs. 5.9% for systolic LAA volume; and 7.7 vs. 17.1% for LAA ejection fraction). No significant differences were found between 2D-S and 3D-S measurements.

**Conclusions:**

In this study, we introduced a fully 3D approach for LAA characterization, allowing the simultaneous assessment of LAA function and geometry. The proposed approach could be used to improve the patient selection and the best sizing of the device for LAA closure and to allow a patient-specific 3D printing.

## 1. Introduction

The left atrial appendage (LAA) is a site responsible for 90% of thrombus formation in patients with atrial fibrillation (AF) [1]. Thrombus formation is associated with reduced LAA contractility as LAA flow stasis predisposes to stagnation and thrombosis. Hence, early evaluation of LAA function could be useful to stratify the risk in patients with AF. In particular, LAA function evaluation was demonstrated to reduce the risk in patients with ischemic stroke and transient ischemic attack (TIA) [2]. In the case of AF and contraindications to anticoagulation therapy, percutaneous LAA closure represents a treatment strategy to reduce the cardioembolic risk [3]. Hence, accurate evaluation of the LAA morphology could also be useful in the preprocedural planning, allowing the selection of the most appropriate device size [4, 5].

Current imaging protocols for assessment of LAA anatomy and functionality involve the use of multiple detectors computed tomography (MDCT), cardiac magnetic resonance (CMR), and transoesophageal (TEE) or intracardiac (ICE) echocardiography [1, 6, 7]. The use of volumetric techniques, such as MDCT, CMR, and 3D Ultrasound, allows obtaining additional qualitative and quantitative information not otherwise available [7]. In the clinical practice, image analysis for the characterization of LAA is based on the reconstruction of appropriate views and the manual delineation of LAA endocardium. LAA volume is then calculated by the Simpson rule. This approach is followed in the analysis of TEE [8, 9], MDCT [10], and CMR [11] images. Due to the complex anatomy of LAA, the standard procedure previously described could be affected by high inter- and intraobserver variability and could require high processing time.

The aim of this study is to introduce a comprehensive approach based on a full 3D analysis of tomographic dataset able to extract morphologic and functional information in the entire cardiac cycle, reducing operator dependence and optimizing the processing time. The proposed methodology has been validated on MDCT dataset including ten patients with AF.

## 2. Materials and Methods

### 2.1. CCTA Imaging

Images from ten consecutive patients (mean age 79 ± 3 years, 4 females) scheduled for LAA closure procedure were retrospectively analyzed. Informed consent was obtained from each patient. The study protocol conforms to the ethical guidelines of the 1975 Declaration of Helsinki as reflected in a priori approval by the institution's human research committee. The examinations were performed by using a 320-detector scanner, with a multiphasic acquisition (Toshiba Aquilion One, Toshiba, Japan) using iodinated contrast medium. Adjacent axial images were reconstructed with a slice thickness of 1 mm, pixel size 0.419 × 0.419 mm, and retrospective ECG-gating, covering the 0–90% of the RR interval at 10% increments (10 frames).

### 2.2. 3D-S Method

**Figure 1:**
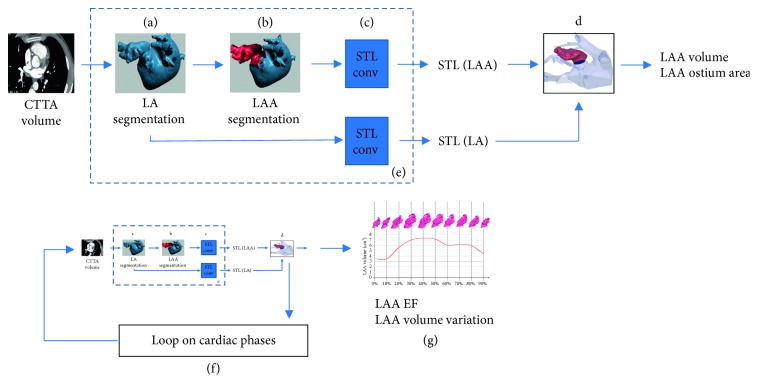
Flow chart of the proposed method. 3D images are processed in segmentation blocks including LA segmentation (a), LAA extraction (b), and conversion of LAA surface in STL model (c). STL model is processed (d) to extract functional LAA parameters. The procedure is iterated among the cardiac cycle (f, g).

**Figure 2:**
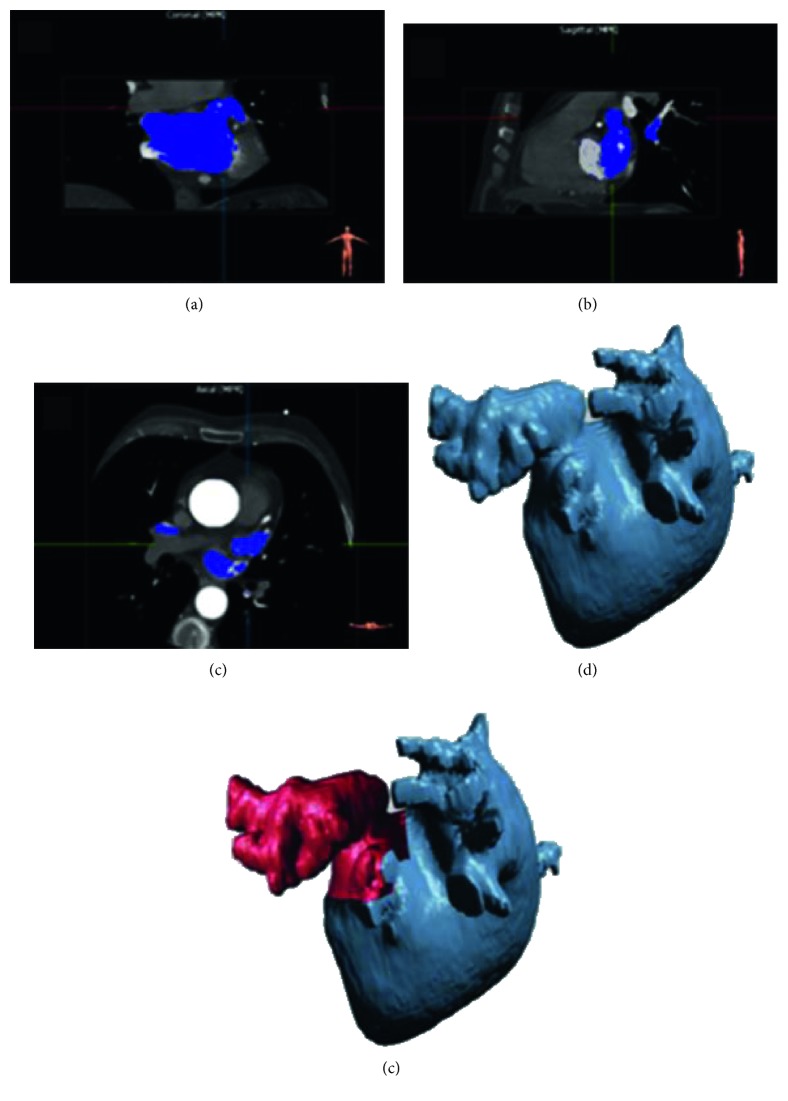
Triplanar views of the LA mask ((a)–(c)). LA surface extracted from LA mask (d). LAA surface extracted from LA surface (e). Circumflex artery and pulmonary vein were removed to improve LAA visualization.

**Figure 3:**
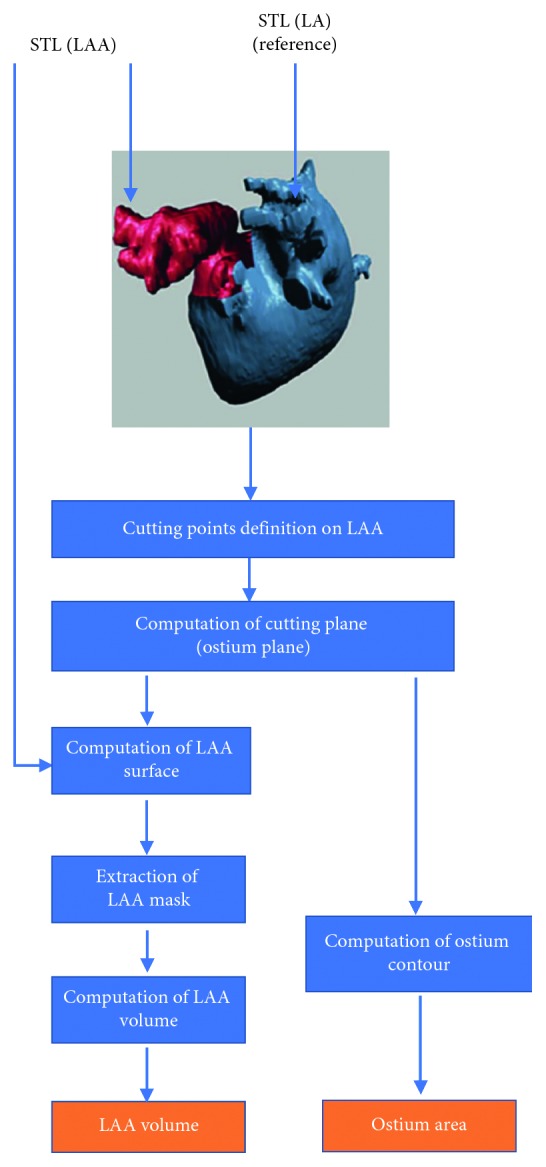
Flow chart of the procedure for geometrical characterization of the LAA, as described in Section 2.2.

**Figure 4:**
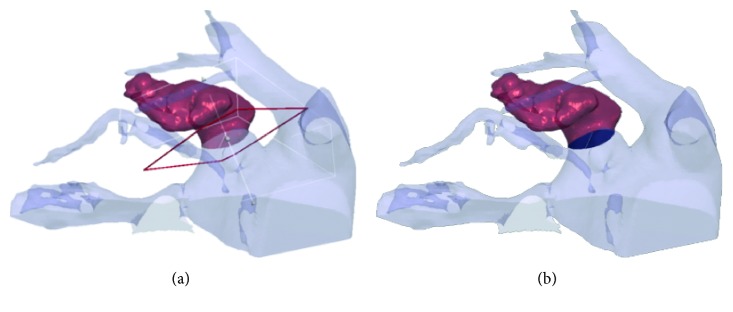
Definition of the cutting plane for LAA ostium identification. (a). Ostium contour defined by the intersection of LAA surface and the cutting plane (b).

The extraction and characterization of dynamic 3D LAA models were obtained by the following procedure that exploits the 3D nature of image data by extracting and processing surfaces (3D-S method). The 3D-S method involves several steps for each phase of the cardiac cycle, as illustrated in Figure 1:

- Segmentation of the CTTA dataset for the 3D LA surface extraction (Figure 1(a)). The LA mask was obtained by applying a threshold-based segmentation procedure on the CTTA dataset. Figures 2(a)–2(c) show the orthogonal views of the LA mask computed from a representative dataset. The initial value of the signal threshold was automatically defined and could be interactively adjusted by the operator to optimize the mask extraction. The LA surface was automatically generated from the mask by an appropriate surface reconstruction algorithm (Figure 2(d)).
- Extraction of the LAA surface from the whole LA surface obtained at step (a) (Figure 1(b)). The LAA surface was manually identified and cropped from LA surface (Figure 2(e)). The manual identification is needed to preserve the whole LAA structure and to remove the unnecessary LA surface parts because the LA surface could include structures in contact or close to LAA surface, as the circumflex artery and the pulmonary vein.
- Conversion of LA and LAA surfaces in STL format (Figure 1(c)). After the LAA cropping procedure, two surfaces were obtained: the LA surface including LA and other structures and the LAA surface. Both the generated surfaces were converted in a portable file format (i.e., STL).
- Measurement of LAA volume and LAA area (Figure 1(d)). In the this step, an ad hoc developed plug-in allowed to perform the measurements of the LAA volume and LAA ostium area, directly from the LAA 3D surface model, including solutions for the reading and the modification of STL imported files and for their three-dimensional visualization through surface rendering and polygonal representations of 3D objects. The complete flow chart of the operations performed in the plug-in is provided in Figure 3. Firstly, a cutting plane was created in correspondence of LAA ostium by selecting cutting points directly on the 3D LAA surface and with the help of circumflex artery and pulmonary vein ridge on the LA surface, that were used as references. The LAA surface and the cutting plane are shown in Figure 4(a), and the final result of the ostium contour defined as the intersection between the LAA surface and the cutting plane is shown in Figure 4(b). The ostium area was automatically evaluated from the ostium contour. The union of the ostium and the part of the LAA surface area up to the cutting plane defined a closed surface encompassing the LAA volume (Figure 4(b)). The corresponding mask was extracted with a voxel size equal to the resolution of the original CTTA, mimicking the standard manual procedure. The LAA volume was automatically calculated by counting the voxels in the mask and multiplying for the voxel size.

It is worth to note that the (a–c) steps (Figure 1(e)) correspond to image processing functions available in several free/commercial software available for research or clinical use, while the (d) step needed the implementation of a specific plug-in able to impost STL models of LAA and to provide the required measurements. 3D models covering the entire cardiac cycle were obtained by iterating the previously described procedure for all cardiac phases (Figure 1(f)). The interactive manual adjustment of the signal threshold is usually needed only for the first frame. Moreover, all the data defined for the previous frame can be used as the starting point for the subsequential processing allowing a reduction of the processing time. The operator can modify, if necessary, the input data (LAA cropping and cutting plane placement) in each cardiac phase. The availability of 3D models for each phase allowed the evaluation of the time volume variation *V*_LAA_(*t*) and the automatic measurement of the LAA ejection fraction (EF_LAA_), using the following equation (Figure 1(g)):

(1) $$\begin{array}{c} {\text{LAA}}_{\text{EF}}=\frac{\max\left[V_{\text{LAA}}\left(t\right)\right]-\min\left[V_{\text{LAA}}\left(t\right)\right]}{\max\left[V_{\text{LAA}}\left(t\right)\right]}. \end{array}$$

### 2.3. Validation and Statistical Analysis

To validate the proposed 3D-S method, standard manual analysis was performed followed by the established clinical procedure (2D-S method) [10, 12]. Transversal views with respect to LAA principal axis were reconstructed from the CTTA images preserving the 1 mm interslice distance by using Osirix MD software (9.0 Version, Pixmeo Sarl, Geneva, Switzerland). The diastolic and systolic frames were manually selected. For both diastolic and systolic phases, the LAA endocardial border was manually delineated on all the transversal slices. LAA trabeculations were considered as a part of the LAA cavity. LAA volume was calculated with Simpson's method by multiplying each manually traced LAA area by the section thickness and summing up the volumes of the separate sections.

The proposed 3D-S method was implemented by using 3Mensio software (9.0 Version, Pie Medical Imaging, Maastricht, The Netherlands) for the (a–c) steps of the procedure (Figure 1(e)), and a custom plug-in software was developed in Matlab (R2016b version, Mathworks Inc., Natick, MA) for the (d) step (measurements of the ostium area and the LAA volume, Figure 1(d)). The custom plug-in was also able to extract LAA functional parameters from LLA volume vs. time curve (Figures 1(f) and 1(g)).

To evaluate the reproducibility and concordance of the 2D-S and the 3D-S methods, two blind operators (interventional cardiologists, more than five years' experience) performed the 2D-S and the 3D-S procedures including only the diastolic and systolic phases as visually defined by the operator. The required processing time was noted for both methods. Ostium area and LAA volume were normalized by BSA calculated by Mosteller formula [13]. Continuous data were expressed as mean ± standard deviation. The interobserver reproducibility was assessed by coefficient of variation (CoV), for both methods. Agreement between methods was assessed by the paired *t*-test.

## 3. Results

**Figure 5:**
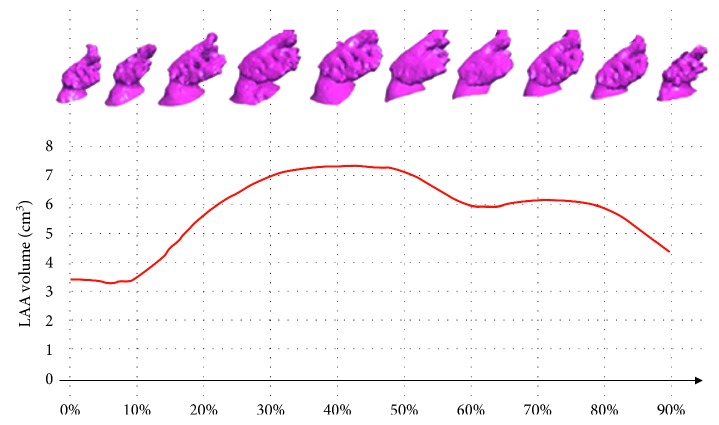
3D LLA models for all cardiac phases and related LAA volume curve.

The 3D-S method was feasible in all cases.  Figure 5 shows the typical results obtained by the procedure. The ten 3D LAA models evaluated in all the cardiac phases are shown, together with the related *V*_LAA_(*t*) curve. The automatic detected diastolic and systolic phases are shown as well. Resulting EF_LAA_ in the presented case was 57%.

**Figure 6:**
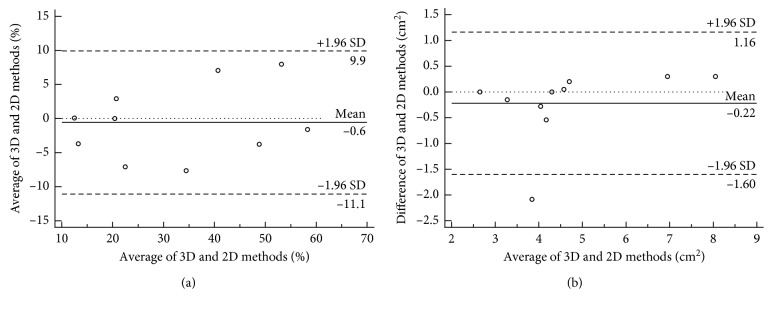
Bland–Altman plots illustrating the relationship between ejection fraction assessed by 2D and 3D methods (a) and diastolic ostium areas assessed by 2D and 3D methods (b).

Table 1 compares the measurements performed by 2D-S and 3D-S methods. A good agreement was found between the 2D-S and 3D-S methods for all measurements. No significant differences were found.  Figure 6 shows Bland–Altman plots related to ejection fraction and diastolic ostium area that are the main parameters used for functional and morphological LAA assessment, respectively.

Reproducibility results are reported in Table 2. CoV was below 10% for all measurements except EF_LAA_ evaluated with the 2D-S method. A good agreement was found between observers for both 2D-S and 3D-S methods. No significant difference between observers was found for all parameters.

The mean processing time for the 2D-S method was 20 ± 6 min; the mean processing time for the 3D-S method was 23 ± 3 min (*p*=0.17). For the 3D-S method, the segmentation phase covered most of the processing time (about 80%), while reconstruction and calculation of LAA indices required about 15% and 5% of the overall processing time, respectively.

## 4. Discussion

Assessment of the LAA function and geometry represents a key issue in the thrombosis risk stratification in AF patients and the planning of percutaneous LAA closure. In the clinical practice, the LAA function is evaluated on original datasets by the 2D-S method and not from reconstructed 3D surfaces as the use of 3D surfaces is expected to introduce loss of information [10]. In the preprocedural planning, the choice of the device is usually done by measurement of the internal LAA diameter [5] or LAA ostium size [14]. However, the use of 3D LAA models was demonstrated to be associated with better preprocedural planning [15], also by the use of printed MDCT 3D models [16, 17].

In this study, we introduced a fully 3D approach (3D-S) for LAA characterization, allowing the contemporary assessment of the LAA function and geometry. The comparison between the proposed 3D-S approach and the standard 2D-S method in the assessment of LAA function parameters revealed a strong concordance (Table 1), proving that the extraction of LAA reliable measurements directly from 3D LAA models is feasible. The interobserver reproducibility of the 3D-S method was better with respect to the 2D-S method (Table 2). Most of the detected variability was correlated with the selection of the LAA cutting plane. The processing time associated with the 3D-S method was slightly longer with respect to the 2D method, but acceptable in the clinical setting. Moreover, if both LAA function and LAA geometry should be assessed, the 3D approach would allow a significant reduction of the image processing time.

The mean EF_LAA_ value obtained in this study (32 ± 18%) was significantly lower compared to the EF_LAA_ value in normal subjects (55 ± 17%), as reported in a previous work [10]. This finding confirmed the reduction of LAA contractile function in AF patients at risk of thrombus formation.

To choose the closure device size and to plan the procedure, the availability of the 3D LAA model only at the end-diastole time is necessary because it is sufficient to consider the cardiac phase with the larger LAA size. However, the availability of models covering the full heart cycle could allow the development of other applications, for example, the LAA compliance characterization using the correlation between the LAA volume and the atrial pressure variation in the time [18].

**Figure 7:**
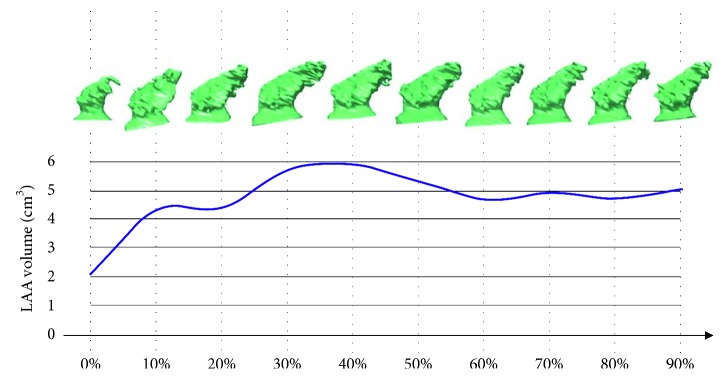
3D LAA models for all cardiac phases extracted from 3D ICE images and the related LAA volume curve. LAA models were extracted by using 3D Slicer software [24].

In this study, the 3D-S method was applied to CTTA datasets, included in our centre protocol for the planning of the LAA closure procedure. Although CCTA was demonstrated to more accurately predict LAA anatomy as compared to echocardiography [19, 20], 3D TEE and ICE are commonly used as image modality for LAA characterization [21]. A possible limit of CCTA is the limited number of cardiac phases that could be reconstructed maintaining an acceptable SNR value. In the present study, ten cardiac phases were reconstructed as proposed in the assessment of whole cardiac function [22] and done in similar studies of LAA function [10, 23]. Echocardiography and cardiac MRI could allow to image the LAA cycle with a better temporal resolution. The proposed 3D approach could be easily extended to other 3D image modalities, such as MR and echocardiography. In fact, the second step of the procedure is totally independent of the used image modality. The first step can be performed with any segmentation software able to define a 3D model of the LA.  Figure 7 shows the 3D LAA surface models created from a 3D intracardiac echocardiography dataset and the corresponding LAA volume measurements calculated with the 3D method herein proposed.

## 5. Conclusions

A fully 3D approach based on the extraction of LAA geometry through the cardiac cycle allows to effectively assess both LAA function and geometry with a better reproducibility in respect to the 2D image analysis approach commonly used in clinical practice. The 3D-S approach could be also useful to optimize the device sizing procedure for LAA closure.

## Figures and Tables

**Table 1:** Comparison between measurements performed by 2D-S and 3D-S methods.

LAA parameter	3D-S	2D-S
Diastolic ostium area (cm^2^)	4.54 ± 1.80	4.77 ± 1.53
Systolic ostium area (cm^2^)	3.58 ± 1.82	3.79 ± 1.69
Diastolic LAA volume (cm^3^)	10.26 ± 5.25	10.55 ± 4.88
Systolic LAA volume (cm^3^)	7.39 ± 3.35	7.46 ± 4.71
Normalized diastolic ostium area (cm^2^/m^2^)	2.63 ± 1.22	2.76 ± 1.08
Normalized systolic ostium area (cm^2^/m^2^)	2.07 ± 1.15	2.20 ± 1.09
Normalized diastolic LAA volume (cm^3^/m^2^)	5.98 ± 3.71	6.19 ± 3.63
Normalized systolic LAA volume (cm^3^/m^2^)	4.32 ± 3.35	4.38 ± 3.27
EF_LAA_	32.19 ± 17.83	32.76 ± 16.46

EF_LAA_: LAA ejection fraction. No significant difference was found for all measurements.

**Table 2:** Reproducibility analysis for 3D-S and 2D-S methods.

LAA parameter	3D-S			2D-S		
	CoV (%)	Bias	CI	CoV (%)	Bias	CI
Diastolic ostium area	2.9	0.07 cm^2^	]−0.36; 0.50[ cm^2^	4.1	0.07 cm^2^	]−0.62; 0.77[ cm^2^
Systolic ostium area	3.8	0.01 cm^2^	]0.28; 0.31[ cm^2^	6.1	−0.13 cm^2^	]−0.73; 0.47[ cm^2^
Diastolic LAA volume	2.4	0.06 cm^3^	]−0.65; 0.77[ cm^3^	5.3	0.10 cm^3^	]−2.05; 2.24[ cm^3^
Systolic LAA volume	2.7	−0.14 cm^3^	]−0.58; 0.03[ cm^3^	5.9	0.04 cm^3^	]−0.87; 0.94[ cm^3^
EF_LAA_	7.7	−0.38%	]−6.28; 5.53[ %	17.1	0.05%	]−12.99; 13.08[ %

## Data Availability

The hospital data used to support the findings of this study have not been made available because there is confidentiality agreement of the hospital.
